# Ameliorative Effects of Loganin on Arthritis in Chondrocytes and Destabilization of the Medial Meniscus-Induced Animal Model

**DOI:** 10.3390/ph14020135

**Published:** 2021-02-08

**Authors:** Eunkuk Park, Chang Gun Lee, Seung Hee Yun, Seokjin Hwang, Hyoju Jeon, Jeonghyun Kim, Subin Yeo, Hyesoo Jeong, Seong-Hoon Yun, Seon-Yong Jeong

**Affiliations:** 1 Department of Medical Genetics, Ajou University School of Medicine, Suwon 16499, Korea; jude0815@hotmail.com (E.P.); dangsunsang@naver.com (C.G.L.); yun41101@ajou.ac.kr (S.H.Y.); tjrwlshh@naver.com (S.H.); wjsgywn0315@ajou.ac.kr (H.J.); danbi37kjh@hanmail.net (J.K.); 2 Department of Biomedical Sciences, Ajou University Graduate School of Medicine, Suwon 16499, Korea; 3 Nine B Co. Ltd., Daejeon 34121, Korea; snsnans@naver.com (S.Y.); jhyesoo921@gmail.com (H.J.); tpohot10@nate.com (S.-H.Y.)

**Keywords:** arthritis, *Cornus officinalis*, loganin, destabilization of the medial meniscus, anti-inflammatory effect

## Abstract

Arthritis is a common inflammatory disease that causes pain, stiffness, and joint swelling. Here, we investigated the ameliorative effects of loganin on arthritis in vitro and in vivo. A single bioactive compound was fractionated and isolated from *Cornus officinalis* (CO) extract to screen for anti-arthritic effects. A single component, loganin, was identified as a candidate. The CO extract and loganin inhibited the expression of factors associated with cartilage degradation, such as cyclooxygenase-2 (COX-2), matrix metalloproteinase 3 (MMP-3), and matrix metalloproteinase 13 (MMP-13), in interukin-1 beta (IL-1β)-induced chondrocyte inflammation. In addition, prostaglandin and collagenase levels were reduced following treatment of IL-1β-induced chondrocytes with loganin. In the destabilization of the medial meniscus (DMM)-induced mouse model, loganin administration attenuated cartilage degeneration by inhibiting COX-2, MMP-3, and MMP-13. Transverse micro-CT images revealed that loganin reduced DMM-induced osteophyte formation. These results indicate that loganin has protective effects in DMM-induced mice.

## 1. Introduction

Arthritis is a medical condition that affects joint inflammation, with pain, swelling, and stiffness of the cartilage [1]. Approximately 250 million people suffer from arthritis with enhancing incidence with aging and obesity concurrently with increasing numbers of joint injuries, indicating that arthritis is a burdensome and prevalent syndrome worldwide [2]. The onset of arthritis is induced by the breakdown of cartilage in the joint, resulting in mechanical stress on the cartilage [3]. Mechanical stress induces the production of interleukin-1 beta (IL-1β) in chondrocytes, which increases the expression of inflammatory factors, such as matrix metalloproteinases (MMPs), cyclooxygenase-2 (COX-2) and prostaglandin E2 (PGE_2_) [4,5]. In addition, nuclear factor-kappa B (NF-κB) plays a critical role in the regulation of MMP-3, MMP-13, and COX-2, which control the production of cartilage extracellular matrix (ECM) and collagen type II alpha 1 chain (Col2a1) [6,7]. Many pharmacological drugs used for the treatment of arthritis have focused on reducing the inflammatory response and include acetaminophen, nonsteroidal anti-inflammatory drugs (NSAIDs), and duloxetine [8,9,10]. Although chemical medications are used to reduce the pathogenesis of arthritis, adverse effects are associated with their long-term use, including constipation, nausea, and vomiting [11].

Herbal plants have been used widely as alternative medicines for the treatment of diseases with fewer side effects than pharmacological medications [12]. Previous studies have revealed that several plant extracts have anti-inflammatory effects, including OA-induced pathogenesis [13,14,15]. In addition, herbal plants contain numerous bioactive compounds, which exert various beneficial effects on diseases [16]. *Cornus officinalis* (CO), a species of dogwood named cornelian cherries, is a deciduous tree belonging to the Cornaceae family that is prevalent in East Asia [17]. CO has been used as a traditional medicine for the treatment of diabetes, hypertension, and renal failure [18,19,20]. A recent study revealed that CO inhibited inflammation by reducing nitric oxide, PGE_2_, and NF-κB activation [21]. In addition, pharmaceutical bioactive components derived from CO extract have been identified, including loganin, morroniside, caffeic acid, and ursolic acid [22]. Loganin, one of the most abundant compounds in CO extract, has been reported to be an anti-inflammatory agent by reducing reactive oxygen species and cytokines [23,24] and inhibiting inflammatory responses by attenuating NF-κB downstream signaling [25,26].

The aim of this study was to identify bioactive compounds in CO extract with the ability to ameliorate inflammation-mediated arthritis. The CO extract was fractionated to isolate a single compound. Next, the protective effects of loganin isolated from CO extract on arthritis were examined in primary chondrocytes in vitro and destabilization of the medial meniscus (DMM) mouse model in vivo.

## 2. Results

### 2.1. CO Extract and Loganin Inhibit IL-1β-Mediated Inflammation in Mouse Primary Chondrocytes

**Figure 1:**
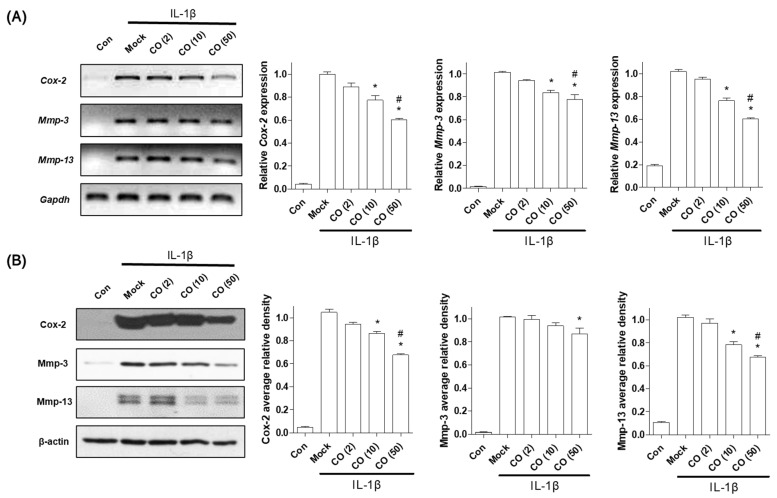
Inhibitory effect of *Cornus officinalis* (CO) extract on chondrocyte inflammation. Mouse primary chondrocytes were exposed to interleukin-1 beta (IL-1β) and treated with CO extract (2, 10, and 50 μg/mL) for 48 h. (**A**) mRNA expression levels of mouse COX-2, MMP-3, and MMP-13 were assessed by reverse-transcriptase polymerase chain reaction (RT-PCR, left) and quantitative RT-PCR (qRT-PCR, right). *Gapdh* was used as an internal control. (**B**) Cell lysates were subjected to Western blot analysis to evaluate mouse COX-2, MMP-3, and MMP-13 (left). β-actin was used as an internal control. The representative image is presented in the left panel, and the densities of three independent experiments are quantified and illustrated in the right panel. The bar graph was analyzed using one-way ANOVA (Tukey’s honest significant difference post-hoc test, analysis of variance). * *p* < 0.05 vs. Mock, ^#^ *p* < 0.05 vs. CO (2). Con = control; Mock = non-treated; CO = CO extract.

To examine the inhibitory effect of CO extract on inflammation, we investigated the inflammatory response induced by IL-1β (1 ng/mL) treatment in chondrocytes. Primary cultured chondrocytes were co-incubated with CO extract (2, 10, and 50 μg/mL) for 48 h. The CO concentration used in this study did not induce any cellular toxicity (Figure S1A). CO treatment reduced both mRNA and protein levels of COX-2, MMP-3, and MMP-13 in a dose-dependent manner (Figure 1), indicating that CO extract inhibited IL-1β-induced inflammation in mouse chondrocytes.

**Figure 2:**
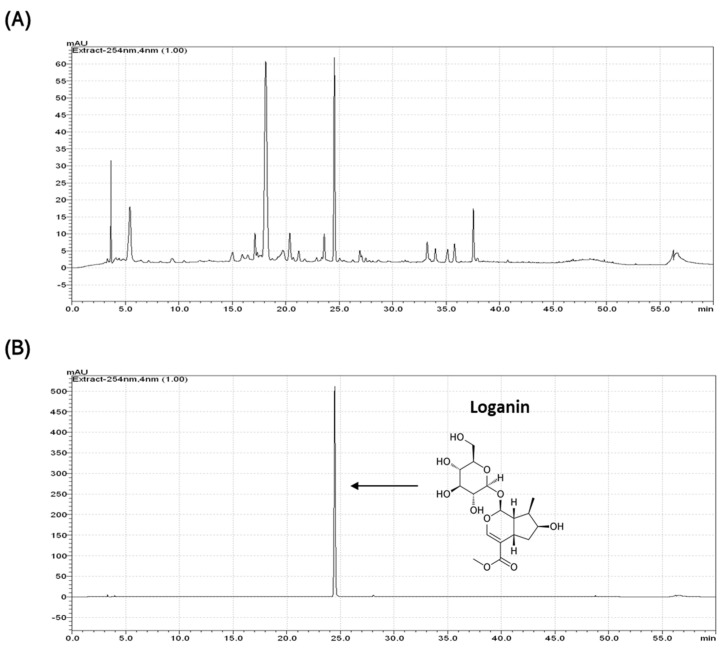
HPLC analysis of (**A**) total CO extract and (**B**) standard loganin compound.

A previous study demonstrated that the CO extract contains different bioactive compounds [27]. We isolated the major components from the CO extract to identify the protective effect on chondrocyte inflammation. HPLC fingerprinting of the CO extract is shown in Figure 2A. Single bioactive compound(s) were fractionated by sequential fractionation using hexane, dichloromethane, ethyl acetate, butanol, and methanol solvents (Figure S2), and the anti-inflammatory effects were evaluated by the expression of COX-2, which is a sensitive marker for inflammation. A significant reduction in COX-2 expression was observed in the butanol fraction, which was further fractionated into six different sub-fractions (Figure S3). Finally, a specific bioactive compound, loganin, was selected and confirmed by comparison with a commercial standard compound (Figure 2B).

**Figure 3:**
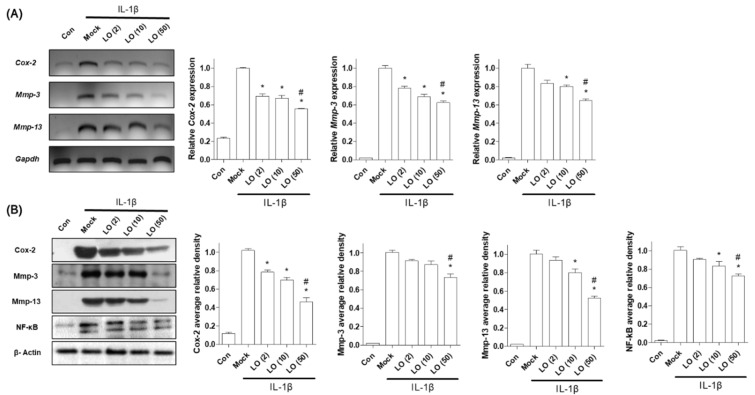
Loganin inhibits IL-1β-induced inflammation in mouse primary chondrocytes. Mouse primary chondrocytes were incubated with IL-1β (1 ng/mL) and loganin (2, 10, and 50 μM) for 48 h. (**A**) mRNA expression levels of mouse COX-2, MMP-3, and MMP-13 were assessed by RT-PCR (left) and qRT-PCR (right). *Gapdh* was used as an internal control. (**B**) Cell lysates were subjected to Western blot analysis to evaluate mouse COX-2, MMP-3, MMP-13, and NF-κB (left). β-actin was used as an internal control. The representative image is presented in the left panel, and the densities of three independent experiments are quantified and illustrated in the right panel. The bar graph was analyzed using one-way ANOVA (Tukey’s honest significant difference post-hoc test, analysis of variance). * *p* < 0.05 vs. Mock, ^#^ *p* < 0.05 vs. LO (2). Con = control; Mock = non-treated; LO = loganin.

**Figure 4:**
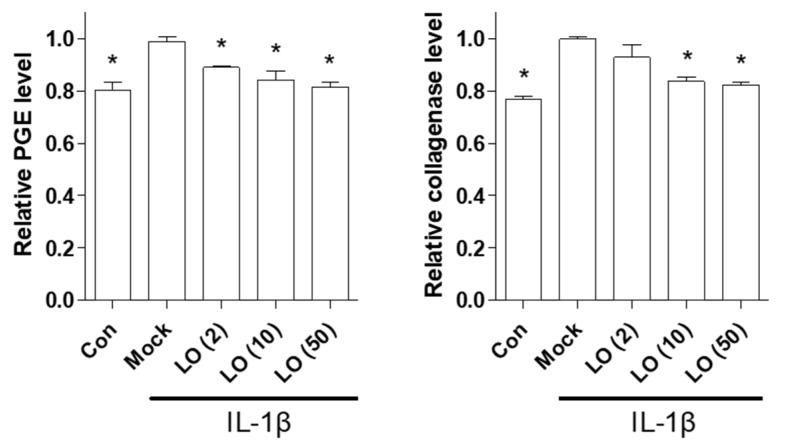
Loganin prevents IL-1β-induced PGE_2_ and collagenase production in mouse chondrocytes. Mouse primary chondrocytes were treated with IL-1β (1 ng/mL) and loganin (2, 10, and 50 μM) for 48 h. The cell supernatants were analyzed using PGE_2_ and collagenase ELISA assay kits. The results from triplicate experiments were analyzed by one-way ANOVA (Tukey’s honest significant difference post-hoc test, analysis of variance). * *p* < 0.05 vs. Mock. Con = control; Mock = non-treated.

Next, we examined the anti-inflammatory effects of loganin on chondrocytes. Inflammation was induced in primary cultured mouse chondrocytes by IL-1β, and cells were incubated with different concentrations of loganin (2, 10, and 50 μM). Loganin treatment did not alter the viability of mouse primary chondrocytes (Figure S1B). However, loganin treatment decreased the expression of inflammatory factors, including COX-2, MMP-3, MMP-13, and NF-κB (Figure 3). In addition, PGE_2_ and collagenase secretion in IL-1β-induced cells was inhibited by loganin treatment (Figure 4). These results indicate that loganin isolated from CO extract has a protective effect on chondrocyte inflammation.

### 2.2. Oral Administration of Loganin Ameliorates DMM-Induced Arthritis In Vivo

**Figure 5:**
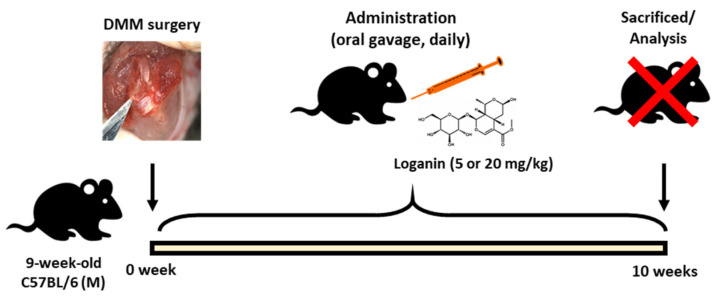
Schematic diagram of the animal study.

**Figure 6:**
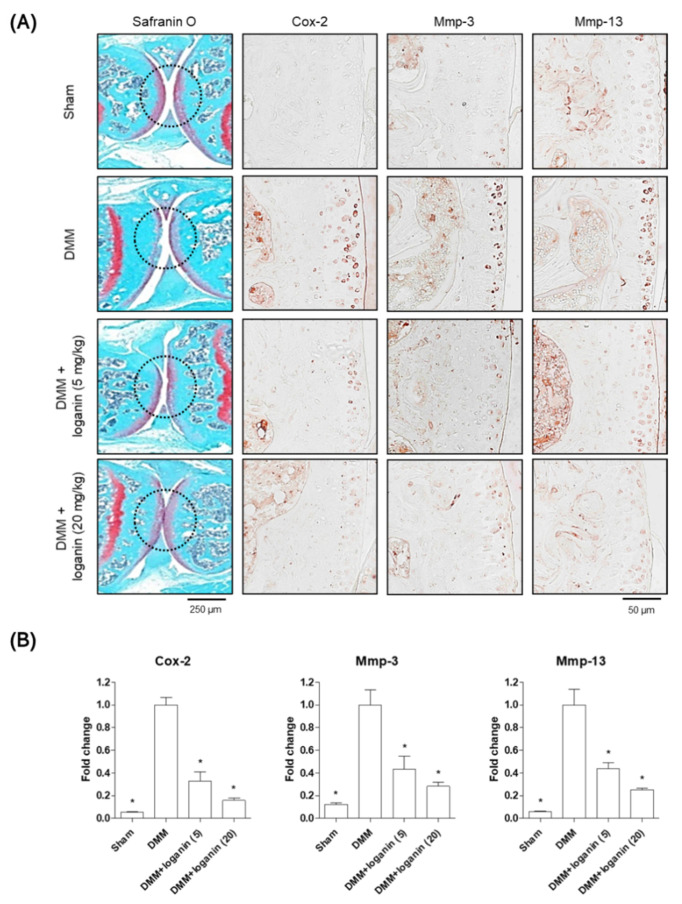
Loganin ameliorates DMM-induced articular cartilage degeneration in mouse cartilage. (**A**) Representative safranin O and IHC images of tibial subchondral bone in DMM surgery mice following loganin (5 and 20 mg/kg/day) administration were visualized under a light microscope (*n* = 5 per group). Sham = sham-operated. Scale bar: 250 μm (Safranin O), 50 μm (IHC). (**B**) Quantification of IHC images in the mouse articular cartilage were analyzed using one-way ANOVA (Tukey’s honest significant difference post-hoc test, analysis of variance). * *p* < 0.05 vs. DMM. Sham = sham-operated; DMM = destabilization of medial meniscus.

**Figure 7:**
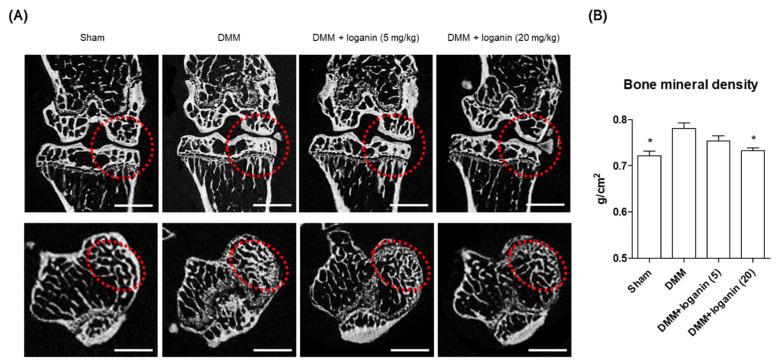
Loganin prevents DMM-induced tibial subchondral osteophyte formation in mice. (**A**) Representative 2D-reconstructed images of tibial subchondral bone in DMM surgery mice following loganin (5 and 20 mg/kg/day) administration or in sham-operated mice obtained by micro-CT analysis (n = 5 per group). Sham = sham-operated. Scale bar: 1 mm. (**B**) Bone mineral density (BMD) of proximal tibial epiphysis was analyzed using one-way ANOVA (Tukey’s honest significant difference post-hoc test, analysis of variance). * *p* < 0.05 vs. DMM. Sham = sham-operated; DMM = destabilization of medial meniscus.

**Figure 8:**
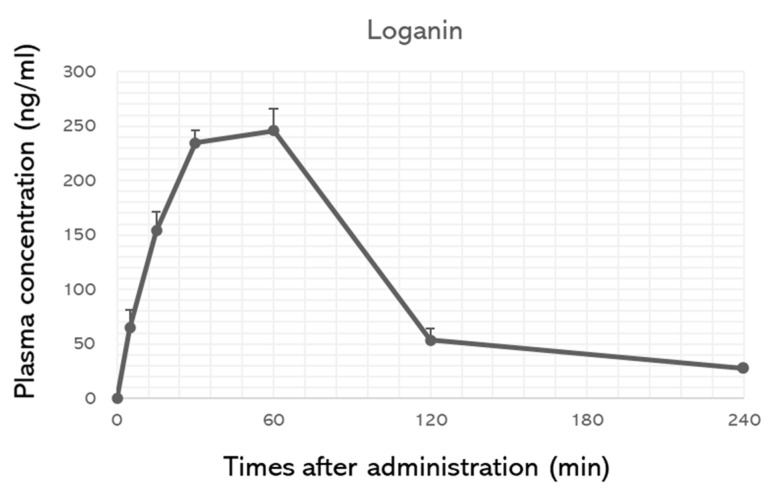
Mean plasma concentration-time curve of loganin in mice after administration of a single dose (5 mg/kg). Data are presented as the mean ± SEM (*n* = 3).

After the in vitro tests, the anti-inflammatory effects of loganin were evaluated in a mouse model of DMM-induced arthritis in vivo. The left medial meniscus of the mice was surgically removed, and loganin (5 and 20 mg/kg/day) was administered daily by oral gavage (Figure 5). After 10 weeks of loganin treatment, arthritis was evaluated histologically by safranin O staining; immunohistochemistry of COX-2, MMP-3, and MMP-13; and micro-computed tomography (micro-CT). DMM-induced subchondral bone plate degeneration was observed with increased positive staining for COX-2, MMP-3, and MMP-13. However, loganin protected against DMM-induced cartilage damage and inhibited arthritic inflammatory factors in the subchondral region of the cartilage (Figure 6). Micro-CT analysis revealed DMM-induced sclerosis in the subchondral bone plate. However, loganin treatment protected the formation of tibial subchondral osteophytes in mice with DMM-induced arthritis (Figure 7). These results suggest that loganin administration ameliorated subchondral bone destabilization by reducing the inflammatory response of chondrocytes. This indicated that loganin inhibits articular cartilage degeneration in vivo. In addition, the loganin (5 mg/kg) plasma clearance test revealed that the concentration of loganin peaked at 60 min and was eliminated within 240 min (Figure 8), suggesting that loganin may have pharmacological properties as a protective agent.

## 3. Discussion

This study describes the anti-inflammatory effects of a single bioactive compound, loganin isolated from CO extract, on arthritis using in vitro and in vivo methods. Loganin is a popular iridoid glycoside found in a variety of herbal plants, including *Alstonia boonei*, *Strychnos nux-vomica*, and *Cornus officinalis* [28]. The loganin content of each plant extract can differ significantly depending on the region, climate, and cultivation procedure [29]. Thus, phytochemical constituents should be considered to examine biological activity and support the traditional use of these species [30]. In this study, HPLC analysis revealed that the CO extract contained loganin as a major component responsible for anti-inflammatory agents; thus, the anti-arthritic effect of the CO extract was exerted by loganin.

Chondrocytes localize in the ECM of the articular subchondral region, which is responsible for maintenance of the superficial layer in cartilage [31,32]. Inappropriate mechanical stress caused by synovial-fluid reduction and ECM degradation induces cartilage destruction, leading to the secretion of inflammatory cytokines, such as IL-1β, in chondrocytes [33]. IL-1β-mediated inflammation in articular chondrocytes is initiated by activation of the NF-κB signaling cascade [34]. IL-1β binds to either IL-1 receptor 1 or Notch 1 in the cell membrane and stimulates NF-κB-dependent downstream signaling, such as COX-2, MMP-3, and MMP-13 transcriptional activity [35]. In addition, IL-1β promotes PGE_2_ and collagenase type 2 secretion, which play a major role in the regulation of ECM degradation during arthritis development [36]. Therefore, controlling IL-1β-mediated inflammation is crucial for inhibiting arthritis-induced pathogenesis. In the present study, loganin prevented IL-1β-induced inflammation in mouse primary cultured chondrocytes through the NF-κB-dependent downregulation of COX-2, MMP-3, MMP-13, PGE_2_, and collagenase expression. These results indicate that loganin exerts an anti-inflammatory effect in primary cultured chondrocytes.

The DMM mouse has been used widely as an experimental animal model for investigating human arthritis [37]. DMM is a mechanical surgery on the knee joint that induces inflammation in the articular cartilage [38]. Continuous mechanical burden and inflammation of the cartilage leads to destruction of the subchondral region with osteophyte development, which is characterized by an increase in subchondral bone density in arthritis development [39].

Our study revealed that the DMM-induced inflammation and destruction in the subchondral region of cartilage with osteophyte formation were prevented following loganin administration. The oral administration of loganin described in this study was characterized by a non-invasive therapeutic approach in comparison to that of previous reports [40], which regarded loganin as an oral therapeutic agent against osteoarthritis. In addition, regarding the pharmacologic efficacy and drug bioavailability, metabolomic profiles, such as plasma concentration, should be used to evaluate drug properties [41]. The plasma concentrations of loganin were eliminated within 4 h after oral administration of loganin. These results suggest that loganin might be a putative pharmacological agent for the prevention of arthritis.

## 4. Materials and Methods

### 4.1. Extraction and Identification of Loganin from CO Extract

CO fruits were obtained from Icheon and Yangpyeong (Gyeonggi-do, Korea). An ethanol extract of CO was evaporated, suspended in H_2_O, and then partitioned successively using hexane (1.6 g), dichloromethane (1.9 g), ethyl acetate (5.2 g), and butanol (40 g). The butanol extract was subjected to chromatography using a Diaion HP-20 gel column (Sigma, St. Louis, MO, USA) and a gradient H_2_O–methanol solvent system (0, 20, 40, 60, 80, and 100% MeOH). Subfractions were subjected to preparative high-performance liquid chromatography (HPLC; Agilent Technologies, Santa Clara, CA, USA) and eluted with MeOH-H_2_O to obtain the compound. Commercial loganin was obtained from Sigma-Aldrich (St. Louis, MO, USA) and dissolved in distilled water for use in the in vitro and in vivo experiments.

### 4.2. Mouse Primary Chondrocyte Experiment and Cell Supernatant Analysis

Primary chondrocytes from the knee joints of 5-day-old mice were isolated using collagenase type II, as previously described [42]. Cells (3 × 10^5^ cells/well) were seeded in 6-well plates and maintained in Dulbecco’s modified Eagle’s medium (Invitrogen, Carlsbad, CA, USA) containing 10% fetal bovine serum and 1% penicillin/streptomycin (Invitrogen). To assess cell viability, cells were incubated in 96-well plates and treated with different concentrations of CO extract (2, 10, and 50 μg/mL) or loganin (2, 10, and 50 μM) for 48 h. Cell viability was assessed using a water-soluble tetrazolium salt (WST) assay following the manufacturer’s instructions (Donginbiotech, Seoul, Korea). The absorbance was measured at 450 nm using an iMark™ microplate absorbance reader (Bio-Rad, Hercules, CA, USA). PGE_2_ and collagenase levels in the cell supernatant were determined using a Prostaglandin E2 Parameter Assay Kit (R&D Systems, Minneapolis, MN, USA) and a EnzChek™ Gelatinase/Collagenase Assay Kit (Invitrogen), respectively, following the manufacturer’s instructions.

### 4.3. Western Blot Analysis and Immunohistochemistry

Cells were lysed with radioimmunoprecipitation (RIPA) buffer (BIOSESANG, Seongnam, Gyeonggi-do, Korea) containing protease and phosphatase inhibitor cocktails (Roche, Madison, WI, USA). Total lysates were separated by sodium dodecyl sulfate-polyacrylamide gel electrophoresis (SDS-PAGE) and then transferred to a polyvinylidene fluoride membrane (Merck Millipore, Burlington, MA, USA). For immunohistochemistry (IHC), sections (3 μm) of the mouse subchondral bone region were placed on poly L-lysine-coated slides, which were then treated with 3% hydrogen peroxide to eliminate endogenous peroxide activity. Changes in the inflammatory response in chondrocytes and subchondral bone region and inflammatory proteins were analyzed with appropriate primary antibodies, as follows: anti-mouse COX-2 (sc-1745, Santa Cruz, Dallas, TX, USA), anti-mouse MMP-3 (ab52915, Abcam, Cambridge, UK), anti-mouse MMP-13 (ab51072, Abcam, Cambridge, UK), and anti-β-actin (sc-47778, Santa Cruz, Dallas, TX, USA). The densities of three independent results were quantified using ImageJ (Version 1.53g) software (NIH, Rockville, MA, USA).

### 4.4. Reverse-Transcriptase Polymerase Chain Reaction (RT-PCR) and Quantitative RT-PCR

Total RNA was isolated from cultured cells using TRIzol reagent (Invitrogen) according to the manufacturer’s instructions. The quantity and quality of RNA were measured by the optical density at 260/280 nm using a NanoDrop One Microvolume UV-VIS Spectrophotometer (Thermo Fisher Scientific, Waltham, MA, USA). Complementary DNA (cDNA) was synthesized using the RevertAid™ H Minus First Strand cDNA Synthesis Kit (Fermentas, Hanover, NH, USA). RT-PCR was performed using the HiPi Plus 5x PCR Master Mix (ELPIS Biotech, Daejeon, Korea), and qRT-PCR was performed using a CFX Connect Real-Time PCR Detection System (Bio-Rad, Hercules, CA, USA), in a total volume of 25 μL containing 100 ng of cDNA and a SYBR Green I qPCR kit (TaKaRa, Shiga, Japan). The following specific primers were used to evaluate the inflammatory response of chondrocytes: forward 5′-GGT CTG GTG CCT GGT CTG ATG AT-3′ and reverse 5′- GTC CTT TCA AGG AGA ATG GTG C-3′ for mouse COX-2; forward 5′- CTG TGT GTG GTT GTG TGC TCA TCC TAC-3′ and reverse 5′-GGC AAA TCC GGT GTA TAA TTC ACA ATC-3 for mouse MMP-3; forward 5′-TGA TGG ACC TTC TGG TCT GGC-3′ and reverse 5′- CAT CCA CAT GGT TGG GAA GTT CTG-3′ for mouse MMP-13; and forward 5′-TCA CTG CCA CCC AGA C-3′ and reverse 5′-TGT AGG CCA TGA GGT CCA C-3′ for mouse *Gapdh*. Relative gene expression was normalized to that of mouse *Gapdh*, and the mRNA expression values were presented as fold-changes compared to the control. The fold-changes were calculated using the 2^−ΔΔCt^ method (ΔΔCt = ΔCt_control_ − ΔCt_treatment_).

### 4.5. Animal Studies

Nine-week-old male C57BL/6 mice were anesthetized using tiletamine/zolazepam (Zoletil; Virbac Laboratories, Carros, France), and the left medial meniscus was surgically removed. After surgery, mice were treated with loganin (5 mg, 20 mg/kg/day) or sham by oral gavage for 10 weeks. At the end of the experiment, the mice were anesthetized with tiletamine/zolazepam (Zoletil; Virbac Laboratories, Carros, France) and placed carefully on a specimen tray in the same position to measure knee joint bone mineral density using a PIXI-mus bone densitometer with the on-board PIXI-mus software (GE Lunar, Madison, WI, USA). Next, the knee joints were harvested and fixed with 4% paraformaldehyde for 48 h. The tissues were decalcified with 0.5 M EDTA (pH 8.0) for 2 weeks and then embedded in paraffin. For the histological assessment of cartilage damage, 5 μm slide sections were deparaffinized with xylene and stained with safranin O (Sigma). Stained tissues were visualized under a light microscope (Leica, Wetzlar, Germany). To analyze the metabolomic profile of loganin, mice were administered loganin (500 mg/kg) and plasma was collected 0, 5, 15, 30, 60, 120, and 240 min after administration. The plasma samples were stored at −80 °C until analysis. All animal experiments, including primary chondrocyte isolation and destabilization of the medial meniscus, were approved by the Institutional Animal Care and Use Committee of the Ajou University School of Medicine (2016–0062).

### 4.6. Micro-CT Analysis

Transverse micro-CT images of the left knee joint were scanned using a high-energy spiral scan micro-CT (Skyscan 1173, Bruker, Billerica, MA, USA) under conditions of 400 μA (current), 60 kV (voltage), 1280 × 1280 (charge-coupled device camera readout), 400 ms (exposure time), and 360° (rotation step). Two-dimensional axial images were reconstructed using the NRecon software (Bruker) and displayed with a spatial resolution of 8.88 μM by an Inveon Research Workplace (Siemens). To improve slice-by-slice manual tracing of the trabecular bone shapes, axial reformats were conducted.

### 4.7. Statistical Analysis

Data in the bar graphs are presented as mean ± standard error of the mean (SEM). All statistical analyses were performed using GraphPad Prism 5.02 software (GraphPad Software, San Diego, CA, USA). The statistical significance of the values between the groups was evaluated by one-way analysis of variance (ANOVA), followed by Tukey’s honest significant difference (HSD) post-hoc test for correction of multiple comparisons. *p* < 0.05 was considered statistically significant.

## 5. Conclusions

In summary, we examined the ameliorative effects of loganin on arthritis in vitro and in vivo. The bioactive single compound loganin was isolated from CO extract for its anti-arthritis effects. Loganin was shown to inhibit IL-1β-induced inflammation, including COX-2, MMP-3, and MMP-13, and decrease PGE_2_ and collagenase secretion in mouse primary chondrocytes. Oral administration of loganin in a murine model of arthritis revealed that loganin attenuated DMM-induced subchondral cartilage destruction. Our results suggest that loganin may be a potential protective agent against arthritis.

## Data Availability

The data presented in this study are available on request from the corresponding author.

## Supplementary Materials

The following are available online at https://www.mdpi.com/1424-8247/14/2/135/s1, Figure S1: Effects of *Cornus officinalis* (CO) extract and loganin on mouse primary chondrocytes. Primary chondrocytes were incubated with different concentrations of (**A**) CO extract (0, 2, 10, and 50 μg/mL) and (**B**) loganin (0, 2, 10, and 50 μM) for 48 h. Cell viability was assessed by WST assay. Figure S2: Fractionation and isolation of the bioactive component from the CO extract. Figure S3: Relative levels of *Cox-2* expression of the six butanol sub-fractions isolated in Supplementary Figure S2 using primary chondrocytes. Cells were exposed toIL-1β and treated with three different concentrations (1, 5, and 10 mg) for 48 h. Relative levels of *Cox-2* mRNA expression were assessed by qRT-PCR and analyzed using one-way ANOVA (Tukey’s honest significant difference post-hoc test, analysis of variance). * *p* < 0.05 vs. Mock.
