# Supplementary material for: Ameliorative Effects of Loganin on Arthritis in Chondrocytes and Destabilization of the Medial Meniscus-Induced Animal Model
Source: Pharmaceuticals (Basel). 2021 Feb 8;14(2):135. doi: 10.3390/ph14020135 (PMC7914920; doi:10.3390/ph14020135)
Supplement: Supplementary file 1 [file pharmaceuticals-14-00135-s001.pdf]

Ameliorative Effects of Loganin on Arthritis in Chondrocytes and Destabilization of the Medial Meniscus-Induced Animal Model

Eunkuk Park [1,2], Chang Gun Lee [1,2], Seung Hee Yun [1,2], Seokjin Hwang [1,2], Hyoju Jeon [1,2], Jeonghyun Kim [1,2],
Subin Yeo [3], Hyesoo Jeong [3], Seong-Hoon Yun [3] and Seon-Yong Jeong [1,2,3,*]

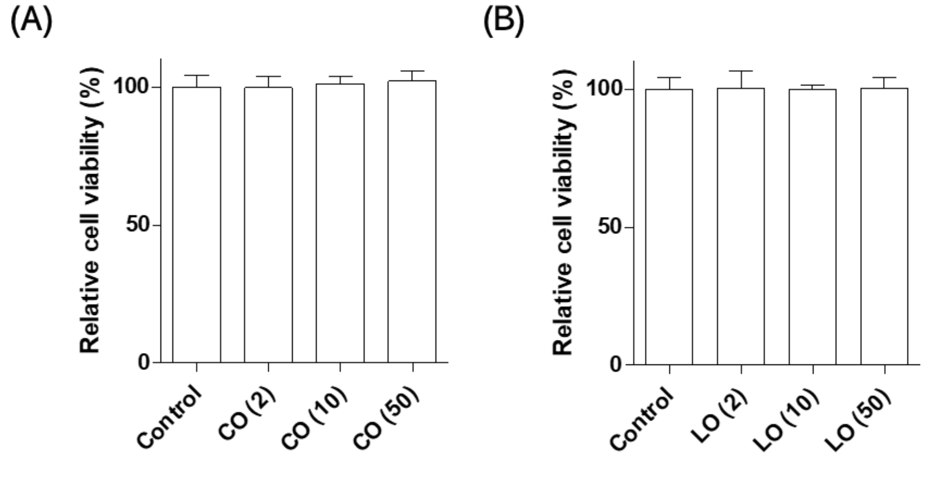

Supplementary Figure S1. Effects of *Cornus officinalis* (CO) extract and loganin on mouse primary chondrocytes. Primary chondrocytes were incubated with different concentrations of (A) CO extract (0, 2, 10, and 50 μg/mL) and (B) loganin (0, 2, 10, and 50 μM) for 48 h. Cell viability was assessed by WST assay.

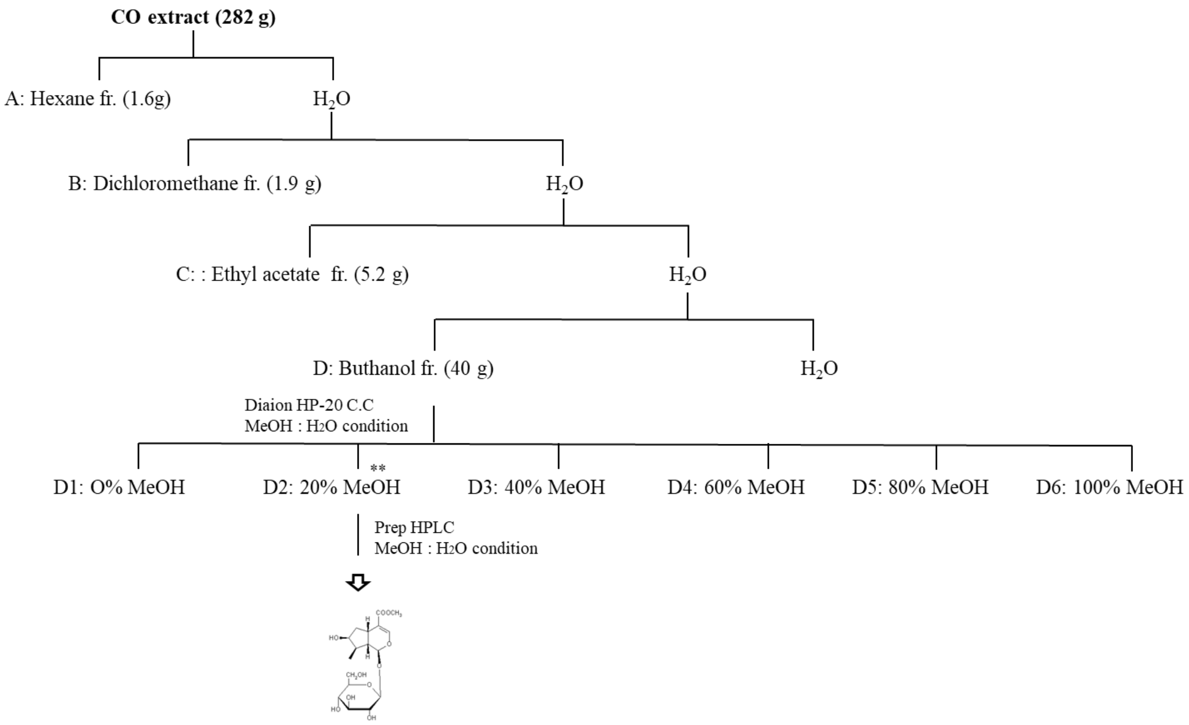

Supplementary Figure S2. Fractionation and isolation of the bioactive component from CO extract.

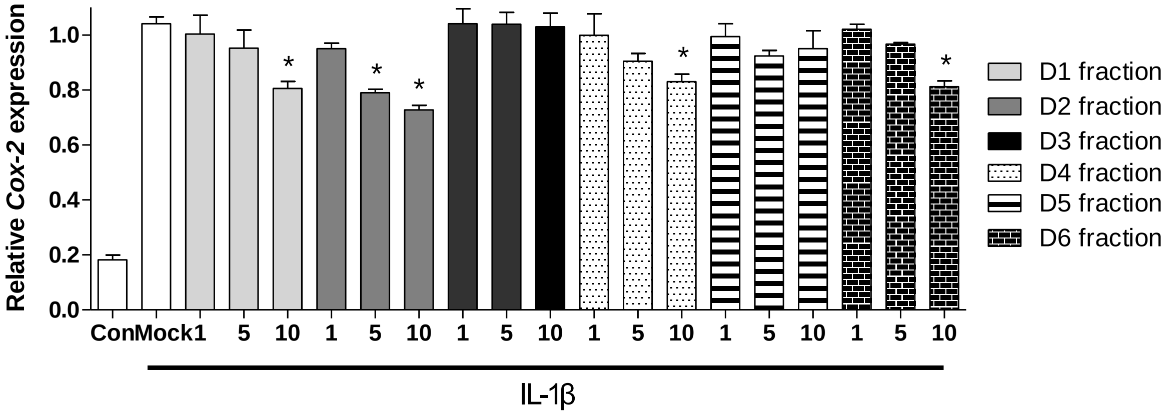

Supplementary Figure S3. Relative levels of *Cox-2* expression of the six butanol sub-fractions isolated in Supplementary Figure S2 using primary chondrocytes. Cells were exposed toIL-1β and treated with three different concentrations (1, 5, and 10 mg) for 48 h. Relative levels of *Cox-2* mRNA expression were assessed by qRT-PCR and analyzed using one-way ANOVA (Tukey's honest significant difference post-hoc test, analysis of variance). $^{*}p < 0.05$ vs. Mock.
